# Insight into the performance of multi-color InGaN/GaN nanorod light emitting diodes

**DOI:** 10.1038/s41598-018-25473-x

**Published:** 2018-05-09

**Authors:** Y. Robin, S. Y. Bae, T. V. Shubina, M. Pristovsek, E. A. Evropeitsev, D. A. Kirilenko, V. Yu. Davydov, A. N. Smirnov, A. A. Toropov, V. N. Jmerik, M. Kushimoto, S. Nitta, S. V. Ivanov, H. Amano

**Affiliations:** 1 Institute of Materials and Systems for Sustainability (IMaSS), Nagoya University, Nagoya, Japan; 2 Center for Integrated Research of Future Electronics (CIRFE), Nagoya University, Nagoya, Japan; 3 Korea Institute of Ceramic Engineering and Technology, Jinju, South Korea; 4 Ioffe Institute, 194021 St. Petersburg, Russia; 5 Department of Electrical Engineering and Computer Science, Nagoya University, Nagoya, Japan

## Abstract

We report on the thorough investigation of light emitting diodes (LEDs) made of core-shell nanorods (NRs) with InGaN/GaN quantum wells (QWs) in the outer shell, which are grown on patterned substrates by metal-organic vapor phase epitaxy. The multi-bands emission of the LEDs covers nearly the whole visible region, including UV, blue, green, and orange ranges. The intensity of each emission is strongly dependent on the current density, however the LEDs demonstrate a rather low color saturation. Based on transmission electron microscopy data and comparing them with electroluminescence and photoluminescence spectra measured at different excitation powers and temperatures, we could identify the spatial origination of each of the emission bands. We show that their wavelengths and intensities are governed by different thicknesses of the QWs grown on different crystal facets of the NRs as well as corresponding polarization-induced electric fields. Also the InGaN incorporation strongly varies along the NRs, increasing at their tips and corners, which provides the red shift of emission. With increasing the current, the different QW regions are activated successively from the NR tips to the side-walls, resulting in different LED colors. Our findings can be used as a guideline to design effectively emitting multi-color NR-LEDs.

## Introduction

Currently, practicable electronics and photonics are based on 2D planar materials, while recently emerging novel 3D materials would be highly desired for the development of future nanoscale systems. For instance, semiconductor nano-crystals are expected to find various applications in, photonics and bioengineering^1^. Due to their relative growth simplicity and size reproducibility compared, e.g., to self-organized quantum dot (QD) arrays, nanorods (NRs) are promising building blocks for nanophotonics. Moreover, NRs present advantageous features for the fabrication of challenging 3D structures. Their low footprint on substrates allows a heteroepitaxy on highly lattice- and thermal-mismatched substrates^2^. Due to the small size of NRs, the strain accumulated in the material during the earliest stage of the growth is released at the sidewalls. Besides, the annihilation and bending of dislocations toward the sidewalls allow the growth of nanocrystals with few or without extended defects. As a result, each NR owns facets of different polarity of high crystalline quality^3^. These facets are very interesting as they possess different surface energy and offer different growth kinetics^4^. Especially, for III-nitride materials which exhibit a hexagonal lattice, different polarization induced fields are inherent for each facet. Therefore, in the case of NR-LEDs, the quantum wells (QWs) grown at each facet are necessarily affected by different local quantum confinement Stark effect (QCSE). Indeed, several group reported multi-emission for such kind of devices^5–8^.

Monolithic integration of RGB nitride-based displays is probably the most important application of the NR-based devices. So far, different growth approaches have been investigated. For instance, catalyst-assisted growth^9^, self-assembled growth^10,11^, selective area epitaxy with^12^ or without pulsing the precursors^13^ have been reported. The device processing of such structures is rather complicate. The common strategy consists in planarization of the device after growth by filling the gaps in the NRs array with an insulating polymer^14^ or directly during the p-GaN deposition by coalescing the rods^15–17^. All of these approaches usually result in more or less leaking devices with insufficient color controllability. The lack of color purity is an additional problem related to the sum of QW emissions coming simultaneously from the different facets. Local inhomogeneity of the shell structure is another deleterious reason which contributes to desaturation of the colors^18–20^. In order to achieve high quality monolithic RGB devices, a deeper insight into the core-shell layer structure and its impact on the optical properties is required. Several reports already presented thorough investigations of the nanoscale properties of the rods^19,21^, while many other were mainly focused on the devices performances and applications^22–25^. However, it is difficult to directly compare the results of such different publications as NRs are often obtained using different growth techniques and exhibit different core-shell structures and crystalline qualities. In addition, due to the lack (or complexity) of characterization techniques at such low scale, it is not rare to find contradictory reports. Therefore, complex exhaustive studies, even if each method is not novel, are important to establish the solid foundation of the NRs technology and to exclude the misleading conclusions from so dispersed data.

In this paper, we demonstrate the successful growth of well-ordered NR arrays and analyze the performance of obtained NR-based LEDs by comparative structural and optical studies done with high spatial resolutions. With detailed data consistently collected from the growth of materials to the device processing and analysis, we draw a critical overview of NR-based LEDs challenge for RGB display application.

## Experimental Section

The NRs were grown by pulsed selective area epitaxy (SAE) in an EpiQuest showerhead MOCVD reactor. At first, a 30 nm-thick SiO_2_ dielectric mask was deposited by reactive sputtering on a commercial n-GaN template (n = 4 × 10^18^ cm^−3^) on silicon (111). An array of 460 nm-wide holes was then opened by a combination of nanoimprint lithography and plasma etching. The NR growth was performed at about 1000 °C under H_2_. The 4.2 Pa trimethyl-gallium (TMGa) and 1.3 × 10^4^ Pa ammonia (NH_3_) were used as precursors and injected alternatively in the growth chamber for 5 and 15 s, respectively. A purge time of 1 s was introduced between each pulse to promote the vertical growth^26^. The pulse sequence cycle was repeated 200 times. Although we did not observe any critical influence of the tetramethyl-silane (TMSi) flow to achieve elongated rods as reported by other groups^27^, we kept a constant Si/Ga ratio of 1.6 × 10^−4^ to ensure a sufficient doping of the GaN core. For the QWs, the carrier gas was switched to N_2_ and the temperature was decreased to 770 °C. The 1.7 Pa trimethyl-indium (TMIn) and 1.8 Pa triethyl-gallium (TEGa) were used as precursors for the QWs. Five QWs were grown and capped with a p-doped GaN layer at 900 °C under H_2_. Bisethylcyclopentadienyl-magnesium (EtCp_2_Mg) was employed as dopant with a Mg/Ga ratio of 2.3 × 10^−3^. Throughout the epitaxial growth, the reactor pressure was kept at 200 Torr.

Structural properties of the samples were first investigated by high-resolution scanning electron microscopy (SEM) by using a JEOL JSM-7001F microscope (resolution 1.2 nm at 30 kV). Transmission electron microscopy (TEM) studies of single NRs placed on a graphene grid were performed by using a Jeol JEM-2100F microscope (accelerating voltage 200 kV, point-to-point resolution 0.19 nm). The optical studies of NRs were done using a micro-photoluminescence (µPL) spectroscopy technique by means of two setups having a resolution of about 1 μm. We exploited Mitutoyo objectives to measure separately a single NR. Detection was done using a liquid nitrogen-cooled charge-coupled detector (CCD). One µPL setup comprises Horiba Jobin Yvon T64000 and LabRAM HR spectrometers equipped by a Linkam THMS600 temperature-controlled microscope stage. In addition, this setup allows measuring the Raman spectra to confirm the high crystal quality of the NRs. The other µPL setup allows carrying out the PL measurements with temporal resolution to investigate the characteristic decay times. This setup is equipped with a single-photon avalanche photodiode (PDM-100-S0E; Micro Photon Devices) and a time-correlated single photon counting (TCSPC) module (SPC-130; Becker & Hickl) for time-resolved PL detection. The cw optically pumped measurements were done with excitation by 405 and 325 nm laser lines. The measurements were performed either at room temperature (RT) or with the temperature variation in a wide range. The time-resolved measurements were done by using a 400-nm line of a femtosecond laser for excitation. The electroluminescence (EL) was recorded under CW current at room temperature with detection normally to the sample surface by an OceanOptics fiber multi-channel spectrometer.

## Results and Discussion

### Characterization of the NR

**Figure 1:**
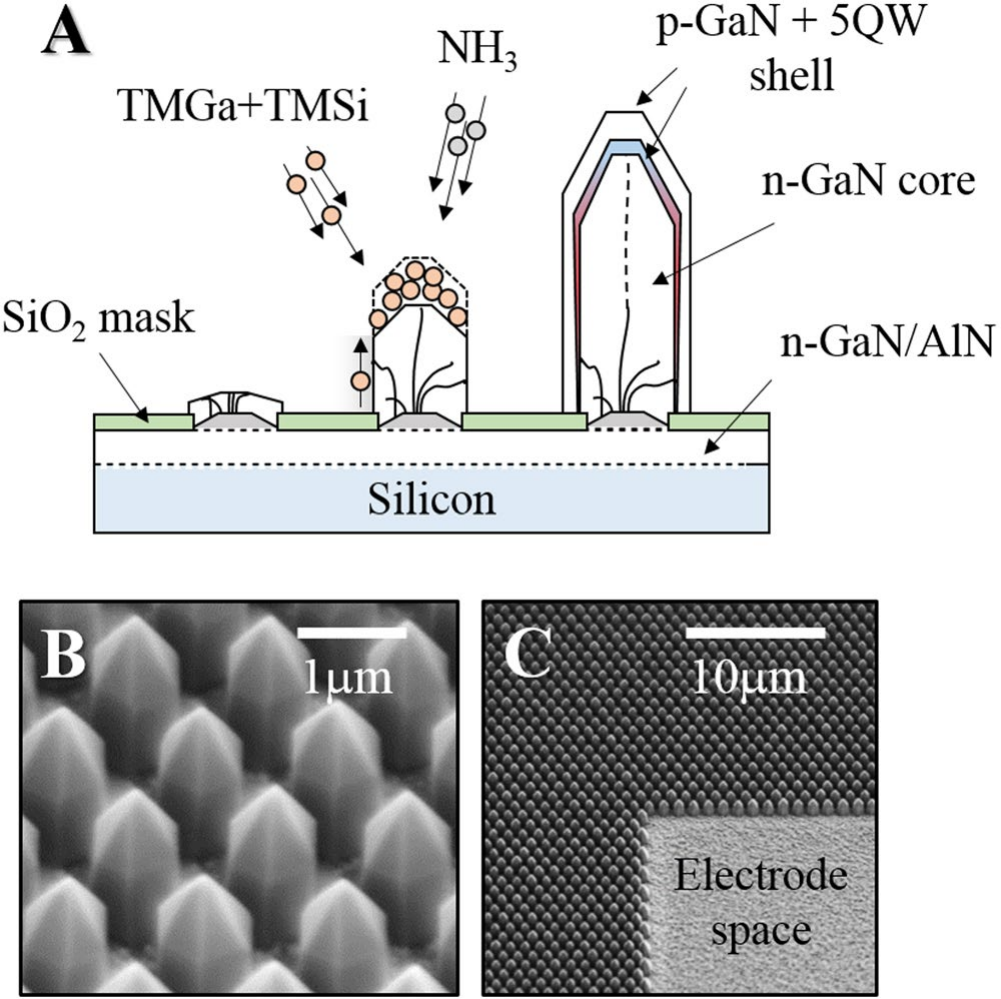
Sketch of the pulsed selective area epitaxy of the n-GaN core followed by a standard 5-InGaN-QW/p-GaN shell deposition (**A**). The resulted NR array is shown on the SEM pictures (**B** and **C**).

Figure 1 demonstrates the fabricated dense array of NRs and illustrates the general principle of the core-shell growth. The typical diameter and the height of the NRs were around 800 nm and 1.5 μm, respectively.

**Figure 2:**
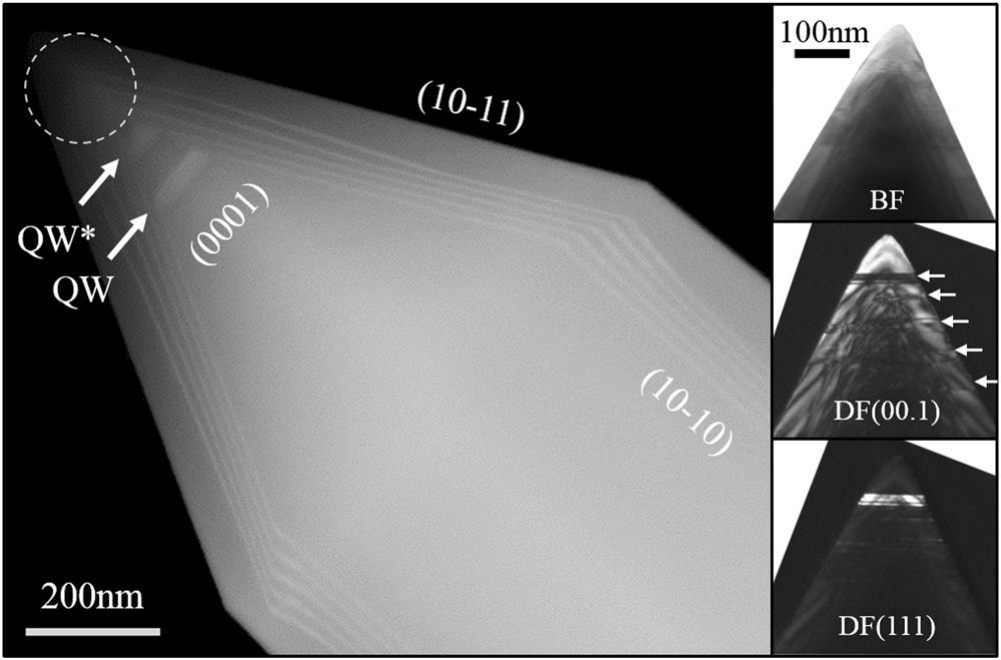
STEM picture of the core-shell layers structure. The right frame shows the presence of BSF in the tip of the NRs. The defects are highlighted under Bright Field (top) and Dark Field contrast (middle and bottom).

Figure 2 shows the top of a NR analyzed by TEM. The slopes on the left image indicate the (10–10) sidewalls and the (10–11) top facets. While the QWs are clearly visible on every facet, their well and barrier thicknesses exhibit distinct increase close to the junction between the top facets and side-walls. They rise dramatically also at the (10–11) facets where clear distortions are seen near the NR tip. Moreover, the (0001) region deviates even more. The first grown (0001) QW is unexpectedly thick, and the second one (denoted as QW* in Fig. 2) is almost as thick as wide, and rather looks like a thick quantum disc. Further (0001) QWs in the upper part of the tip (dotted circle in Fig. 2) are not clearly seen. These QWs, coming from the semi-polar (10–11) facets, converge slightly in the c-direction. The dark-field TEM images taken along [0001] (right side of Fig. 2) show basal plane stacking faults (BSFs) with the presence of cubic material near the tip. They completely mask the top QWs.

In general, the observed structure is consistent with previous reports which noticed different QW thicknesses due to variable deposition rates on different facets^5,28^. Indeed, the QWs were usually half as thick on the (10–11) semi-polar facets than on the (10–10) non-polar sidewalls^3^. Furthermore, the observed QW shape suggests a much higher growth rate along the [0001] direction. On the other hand, it is well known that the growth rates on polar, semi- and non-polar surfaces of planar substrates are quite similar^29,30^. Thus, the large observed difference comes from the specific growth kinetics on differently oriented facets of NRs during a single growth run. This is consistent with the different surface stability at the planes. The (0001) plane is always terminated with high energy dangling bonds, while the (10–10) side facets can form a surface without these dangling bonds, i.e. have a very low energy^4^.

More difficult is the explanation of the situation with the indium incorporation at these facets. On planar substrates, the indium incorporation was reported to be lower on (10–11) than on (0001)^31^, while the emission has been reported at both longer^32^, and shorter^29^ wavelengths as compared with that on (0001). The situation is even more complex for 3D nano-structures like NRs, where the incorporation kinetics on the different planes should be inevitably different. Thus, not only growth conditions, but also size, pitch, and angle of a top cone of the NRs array have a critical impact on the QW homogeneity, as highlighted previously^19,33^. Hence, from so contradictory literature data it is not possible to estimate the indium incorporation and identify the origin of PL bands constituent to the NR emission.

**Figure 3:**
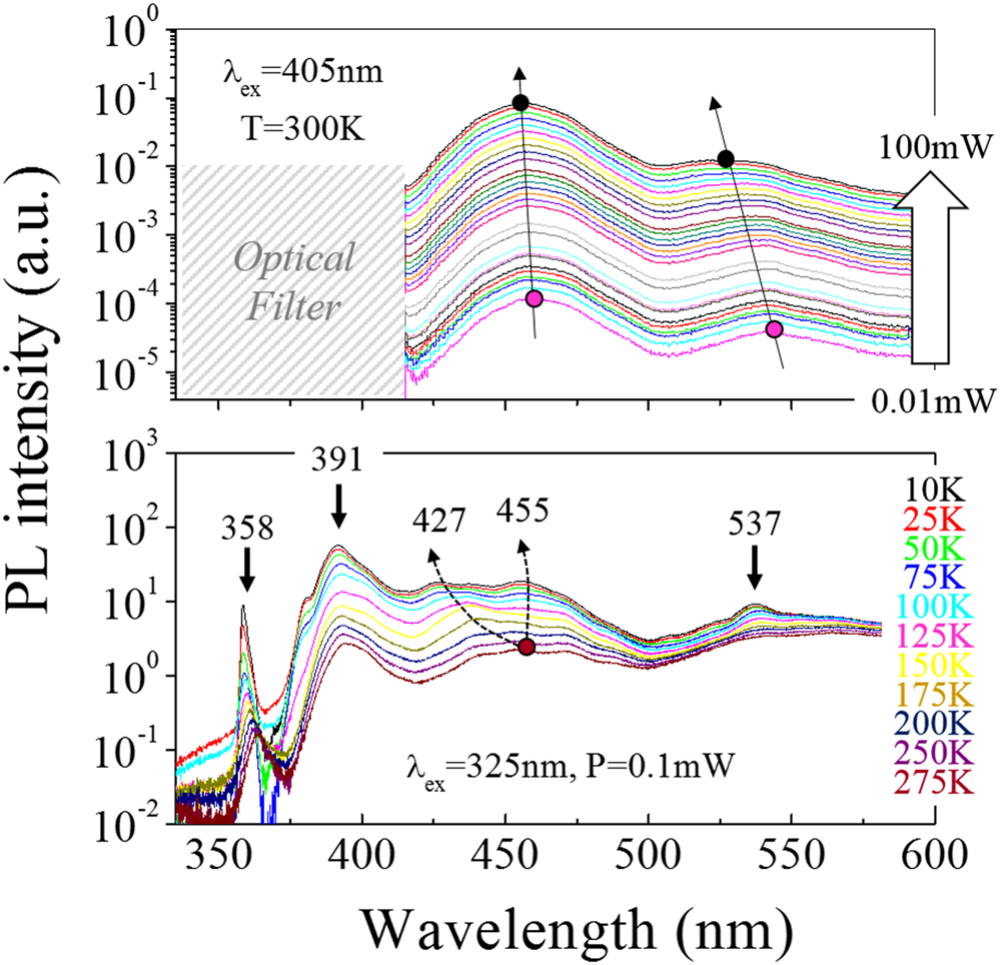
Power (top) and temperature (bottom) dependent PL of the NR based LED structure. The green contribution displays a strong QCSE at high excitation power, while the blue emission splits in two contributions at low temperature.

To shed a light on these important issues we have performed comprehensive PL studies. Figure 3 presents temperature and power dependent PL spectra which allow us to distinguish between the contributions of different QW regions into the NR emission spectra. Four main emission peaks are visible in Fig. 3 (bottom) near room temperature: a clear GaN line near 360 nm, near-UV (390 nm), blue, and green peaks. Besides, a broad background signal centered between 550 nm and 590 nm is observed. This signal is commonly attributed to the so-called yellow band of the GaN^34^. However, we cannot exclude the presence of small contribution(s) coming from the quantum disc-like structures observed by TEM at the very top of the NR.

Let us consider first the blue and green PL peaks. Figure 3 (top) shows a linear relationship between their intensity and the excitation power. Therefore, both these peaks are not defect-related but originate from different QWs grown, presumably, on the different facets. Indeed, due to their finite state density, the typical PL intensity of defects increases linearly at low excitation power before saturating at high excitation power. Furthermore, the blue and green emission bands show the slight and strong quantum confinement Stark effects (QCSE), respectively. At higher excitation power, the photo-generated carriers screen the built-in spontaneous and piezoelectric fields present in the QWs and the conduction and valence bands of the QWs flatten. Consequently, the overlap of the electrons and holes wave functions increases; as a result, the PL energy is blue-shifted and its intensity rises. In addition, the narrower the QW the weaker the intrinsic field and, the less QCSE are expected. This suggests that the green peak, which displays the largest QCSE, originates from the thick QW on the c-plane, while the blue one is a combined signal from the QWs located on the (10–11) facets. At RT, the bands in the blue and green ranges are broad, mainly due to the delocalization of the carriers on each facet. However, at low temperature, the carriers do not have enough energy to escape from the sites of potential fluctuations caused by the small variations of a QW thickness, composition or stress. Hence, the green band sharpens at 537 nm and interestingly, the blue emission splits into two distinct contributions with peaks at 427 nm and 455 nm at 10 K. This splitting can be explained by the signals upcoming from different (10–11) QWs having a variable thickness and morphology, e.g. first three and last two, as can be seen in the TEM image (Fig. 2), or different parts of the QWs.

At these presumptions, we can ascribe the remnant strongest near-UV peak at 390 nm only to the non-polar QWs on the sidewalls of the NRs (m-plane). Several arguments support this assumption. First, the peak does not undergo any clear QCSE at higher excitation power (data are not presented here). It is also interesting to notice this peak exhibits some carrier localization at low temperature, with the appearance of a small peak at shorter wavelength. This suggests a slight QW inhomogeneity along the sidewalls, which is in agreement with Fig. 2. Second, the relative intensity of the near-UV, blue and green peaks match well with the relative surface area of each type QWs. Finally, the carrier lifetime is very short, about 0.5 ns as found by time resolved PL studies, which is rather typical for a non-polar QW. The last argument might be moderated as similar lifetimes have also been reported for QWs on both (10–11) and (10–10) planes^35^. However, this characteristic decay time of 0.5 ns is shortest in our NRs.

### Characterization of the NR-LEDs

**Figure 4:**
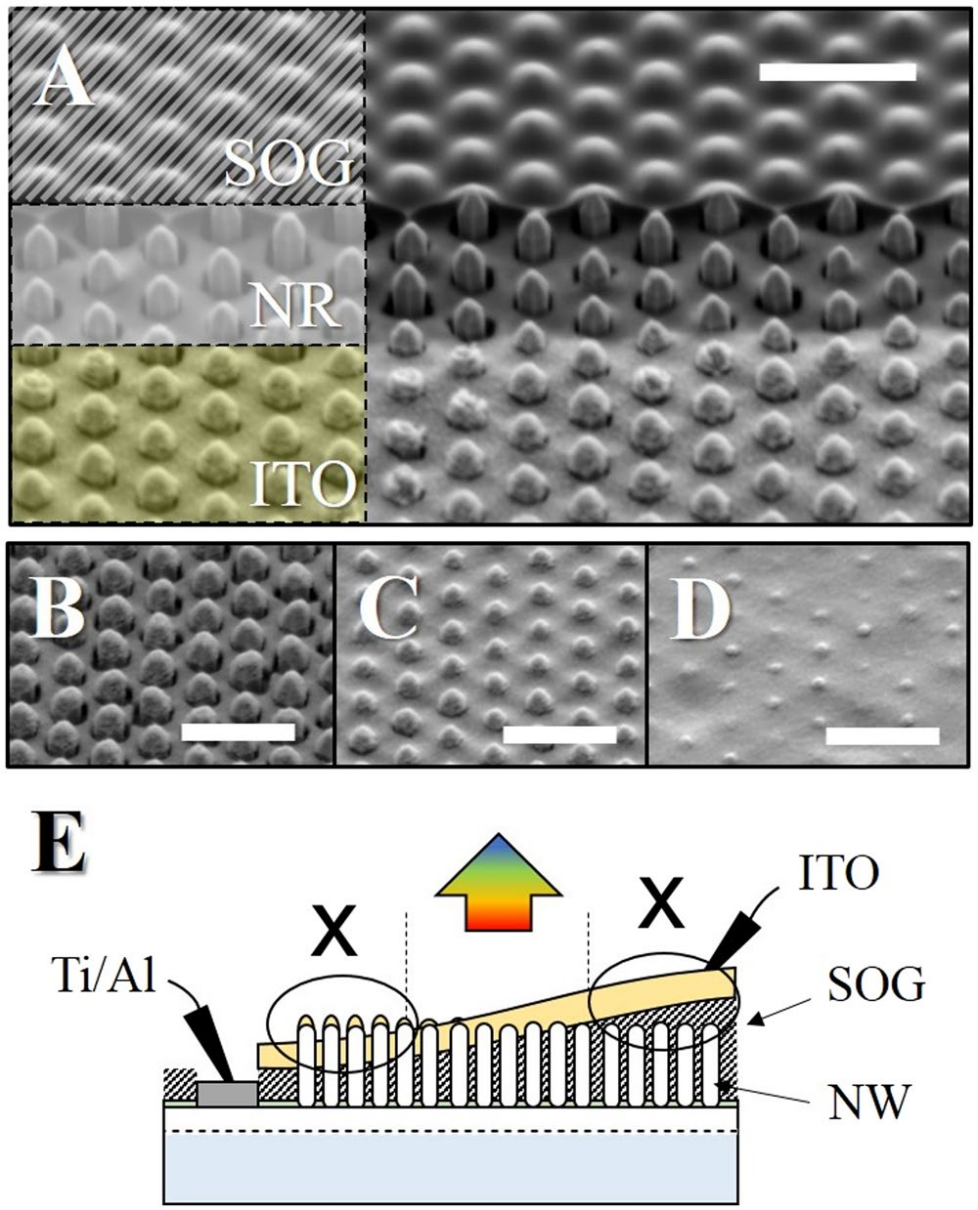
SEM 45° tilted view of the processed device (**A**). For easy understanding, the SOG and ITO layers are shaded similar in the images and in the schematic side view (**E**). NRs in (**B** and **D**) are not electrically connected, due to an over- (**B**) or under-etching (**C**) of the SOG. Picture C shows an area with correct contacts with functional NR-LEDs. The scale bar in each picture is 3 μm.

In order to obtain working NR-LEDs, contacts to the NR are needed. Since the n-GaN template on silicon involves highly insulating AlN and AlGaN layers, the contact to the underlying n-GaN must be on the front. For that purpose, the nano-imprint stamp had dedicated contact areas (see Fig. 1C). The p-side contact requires more efforts. First, the space between NRs was filled with a Spin-On-Glass (SOG) layer by spin-coating. This was necessary to planarize the structure but also to greatly reduce the current leakage. The SOG thickness was adjusted by the spin coater rotation speed to ensure a full coverage of the NRs (compare in Fig. 4B–D).

The SOG layer was cured at 500 °C for 1 h under N_2_. A slow ramping was needed to avoid the formation of holes and cracks which inevitably lead to electrical shortcuts and device failure. The SOG was opened by a standard photolithography and wet etching using a buffered hydrofluoric acid (BHF 1:15). The electrodes were deposited by e-beam evaporation. For the top p-side contact, we chose 150 nm-thick indium-tin-oxide (ITO) while a conventional Ti_5nm_/Al_150nm_ was used as n-side contact (Fig. 4A,E). The ITO electrode was annealed 5 min at 500 °C under O_2_ to improve its transparency (>90%) and conductivity (1.5 × 10^−3^ Ω.cm). The LEDs active region, defined by the ITO surface area, was about 4.5 × 10^−4^ cm^2^. An accurate control of the SOG thickness and its etching rate was critical for a functional device. SEM pictures in Fig. 4B,D show the ITO surface morphology observed respectively at the center and the edge of the sample. Both of the situations result in a non-functional device. In the first case, the top of the rods are electrically disconnected from the base, and in the second case, a thin insulating SOG layer remains between the rods and the ITO. The problem originates from the variation of the SOG thickness, inherent to the spin-coating deposition process. The Fig. 4E summarizes the final device structure as well as the two kind of failure observed.

**Figure 5:**
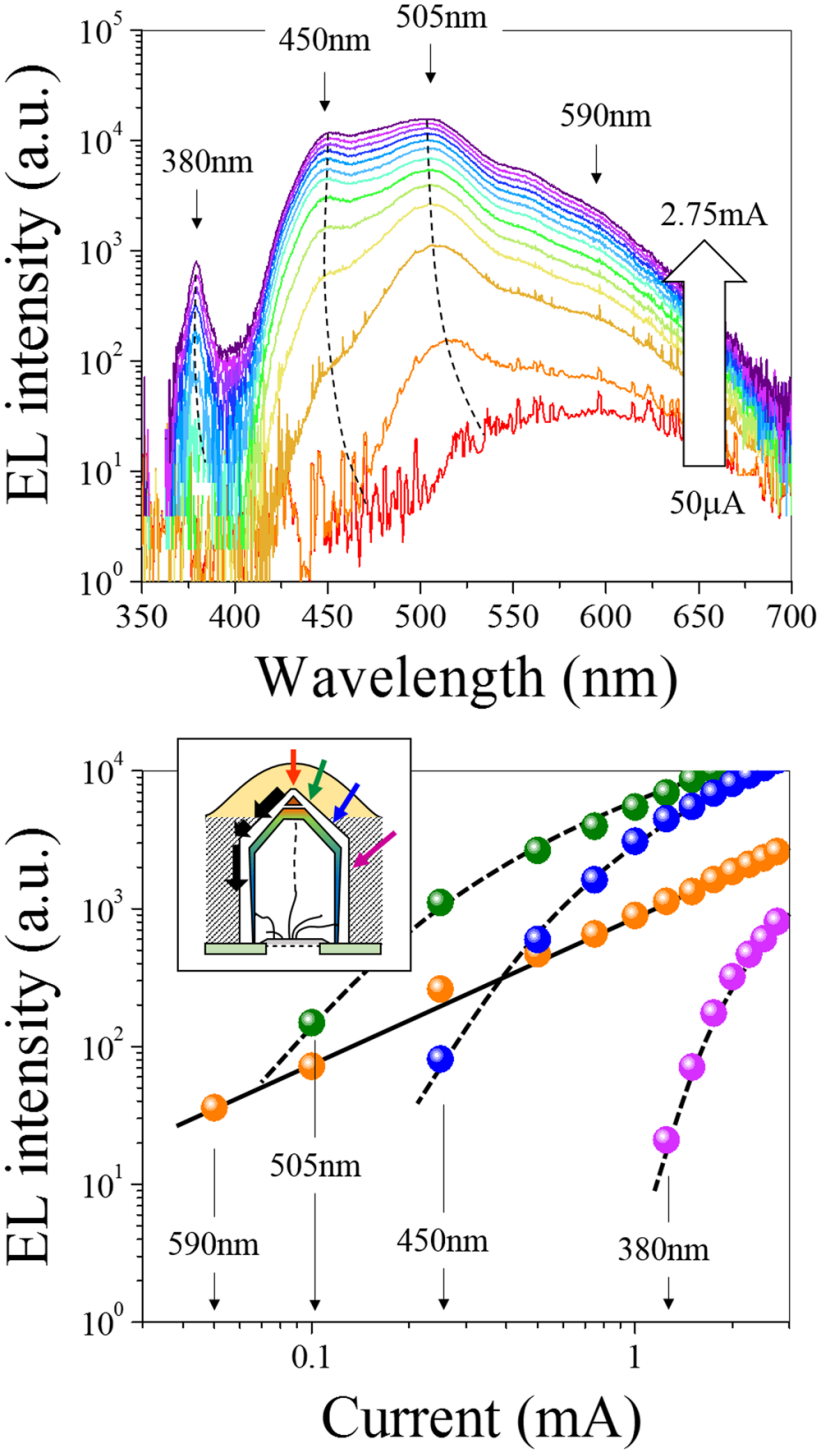
RT-EL spectra recorded at different injection currents (top). Current dependencies of emission intensities at different wavelengths (down). The broad emission is caused by several contributions appearing successively when increasing the current. The inset illustrates the electrical pumping. The spreading of the current along the p-GaN shell leads to the non-uniform excitation of different regions.

The EL spectra strongly changed with the current density (Fig. 5 top). The LED emits weakly around 590 nm at low electrical injection. Pumping at higher intensity broadens the EL, and the maximum shifted toward shorter wavelength. Four distinct contributions successively appear in the orange, green, blue and near-UV range. The energies of the last three peaks are close to those previously observed in the PL spectra. A slight blue shift can be explained by a band filling effect along with the screening of the QCSE at higher carrier injection which shifts the emissions toward shorter wavelengths^36^. The blue shift is strongest for the c-plane related green emission which drifts from about 530 toward 505 nm. The relative intensity of the near-UV, blue, and green bands follows an opposite trend as compared to that characteristic for optical excitation. Now, the main peak comes from the c-plane, while the smaller one, at 380 nm, comes from the m-plane. This difference can be explained by considering the different carrier injection mechanisms between PL and EL. Optical pumping generates carriers within a volume under all the surfaces irradiated, leading to a PL signal greatly affected by the surface area of each facets. However, the EL implies a local pumping when the current passes along a p-conducting layer with the small cross-section area. In the present case, since the ITO electrode is deposited at the very top of the NRs, the EL mainly comes from the tips of the NRs and the GaN cores. With increasing the current density, more carriers reach the semi-polar and non-polar facets far away from the NR top. This explains the successive activation of the different contributions when increasing the current density, as shown in Fig. 5 down). A similar phenomenon has been reported for the same kind of nano-structures^5^. The 590 nm broad band was not so pronounced in the PL as in the EL spectra. Besides, its intensity does not saturate with increasing the current density (Fig. 5, bottom). Thus, this emission does not come fully from deep defect levels. We assume that it can arise partly in the quantum-disk-like regions in the tips of NRs. However, the substantiation of this assumption needs additional investigations.

**Figure 6:**
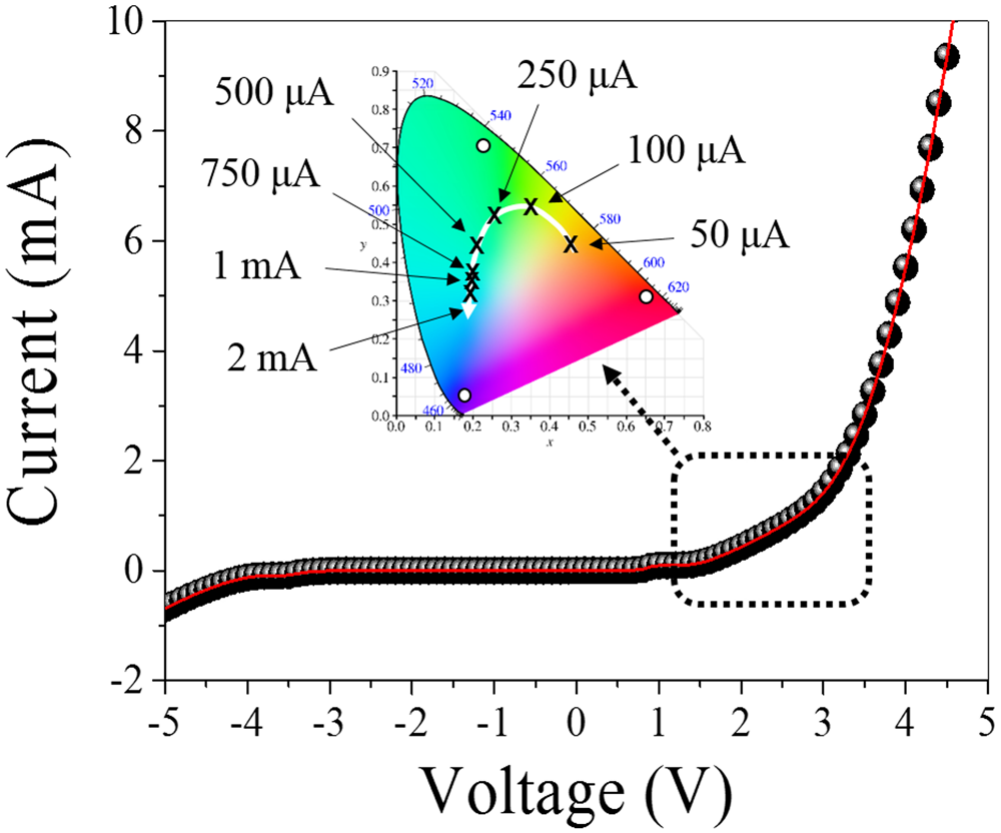
Electrical characteristic of the NR-based LED array. Depending on the current, the colors changes from orange to blue in the CIE xy chromaticity diagram.

The turn-on voltage around 3 V (Fig. 6) is similar to typical blue LEDs. At reverse bias, the structure is weakly leaking, which demonstrates the good quality of the overall processing^15^. However, the series resistance at forward bias is quite high (~120 Ω), mostly due to the bad lateral current spreading of the thin ITO electrode and p-conducting layer. From the drift of the dominant wavelength at different current densities, we calculated the CIE xy color coordinates of the EL spectra and plotted them in the chromaticity diagram (inset in Fig. 6). Depending on the carrier injection, the LED emits from deep orange to blue, covering the visible range. However, the colors are poorly saturated with a chromaticity of about 50, 40, and 68% for the blue, green, and orange colors, respectively. Such color saturation is not yet suitable for display applications, which require highly saturated primary colors (represented by the white dots in the chromaticity diagram of the Fig. 6). An interesting approach to solve this problem would be to control the NRs geometry by tuning locally the selective area pattern size to adjust the total surface of each facet. Enhancing one contribution for the expense of the others should increase the color saturation of the QWs emission. With an appropriate design, it would be possible to realize a high quality multi-color emitting device based on a set of different geometry NRs.

## Summary and Conclusions

We have successfully demonstrated the growth and processing of multicolor NR based LEDs. Using TEM, power and temperature dependent PL, as well as current-dependent EL, we have identified the origin of the different contributions in the LED emission at optical and electrical pumping. These contributions are mainly due to the different growth kinetics on the different crystal planes, which provides the deviations of both QW thickness and indium incorporation. We have observed the generation of orange, green, and blue emissions, although the color saturation still needs improvement. By enhancement of needed contribution from spatially separated NRs of different design it is possible to improve the color purity of the LED and make it less dependent on the current density. This goal can be achieved by developing the pattern for selective area growth, comprising different elementary cells. In general, our findings present a platform for successful implementation of NR arrays with perfect luminescence control for full-color display application.
